# The pH sensitivity of Aqp0 channels in tetraploid and diploid teleosts

**DOI:** 10.1096/fj.14-267625

**Published:** 2015-02-09

**Authors:** François Chauvigné, Cinta Zapater, Jon Anders Stavang, Geir Lasse Taranger, Joan Cerdà, Roderick Nigel Finn

**Affiliations:** *Department of Biology, Bergen High Technology Centre, University of Bergen, Bergen, Norway; ^†^Institut de Recerca i Tecnologia Agroalimentàries (IRTA)–Institut de Ciències del Mar, Consejo Superior de Investigaciones Científicas (CSIC), Barcelona, Spain; and ^‡^Institute of Marine Research, Nordnes, Bergen, Norway

**Keywords:** aquaporin, water transport, permeability lens, cataract

## Abstract

Water homeostasis and the structural integrity of the vertebrate lens is partially mediated by AQP0 channels. Emerging evidence indicates that external pH may be involved in channel gating. Here we show that a tetraploid teleost, the Atlantic salmon, retains 4 *aqp0* genes (*aqp0a1*, *-0a2*, *-0b1*, and *-0b2*), which are highly, but not exclusively, expressed in the lens. Functional characterization reveals that, although each paralog permeates water efficiently, the permeability is respectively shifted to the neutral, alkaline, or acidic pH in Aqp0a1, -0a2, and -0b1, whereas that of Aqp0b2 is not regulated by external pH. Mutagenesis studies demonstrate that Ser^38^, His^39^, and His^40^ residues in the extracellular transmembrane domain of *α*-helix 2 facing the water pore are critical for the pH modulation of water transport. To validate these findings, we show that both zebrafish Aqp0a and -0b are functional water channels with respective pH sensitivities toward alkaline or acid pH ranges and that an N-terminal allelic variant (Ser^19^) of Aqp0b exists that abolishes water transport in *Xenopus laevis* oocytes. The data suggest that the alkaline pH sensitivity is a conserved trait in teleost Aqp0 a-type channels, whereas mammalian AQP0 and some teleost Aqp0 b-type channels display an acidic pH permeation preference.—Chauvigné, F., Zapater, C., Stavang, J. A., Taranger, G. L., Cerdà, J., Finn, R. N. The pH sensitivity of Aqp0 channels in tetraploid and diploid teleosts.

The vertebrate ocular lens is a transparent multifocal organ that refracts and transmits light to a retinal focal point to facilitate color vision (1). It is conserved from jawless lampreys (Hyperoartia) to modern aquatic and terrestrial animals, including sharks and rays (Chondrichthyes), ray-finned fishes (Actinopyerygii), and lungfishes and tetrapods (Sarcopterygii) (2–6). The transparency of the lens arises during embryonic development because of the differentiation of the primary (cortical) and secondary (nuclear) lens fibers, which express high levels of soluble crystallins (7, 8). Subsequently, the differentiating nuclear lens fibers lose all membrane-bound cytoplasmic organelles including mitochondria and nuclei (9). In this state, the lens fibers are avascular and must survive undamaged for the lifetime of the organism (10). In the absence of vasculature, which would otherwise interfere with the transmission of light, an internal microcirculatory system is established between the equatorial epithelial cells and the inner “onion-like” layers of lens fibers (11, 12). This intrinsic circulation is suggested to be established *via* active Na^+^/K^+^ transport, which generates an osmotic gradient facilitating fluid movement through the interstitial space and into the lens fibers *via* membrane-spanning water channels [aquaporins (AQPs)] (12).

AQPs are a ubiquitous class of integral membrane protein that facilitate the transmembrane transport of water, glycerol, or other small solutes and gases (13, 14). Recent studies have shown that the deuterostome superfamily consists of 17 subfamilies (Aqp0–16) with 13 functional subfamilies (Aqp0–12) present in Eutheria (15). Although all 13 members of the eutherian aquaporin superfamily have been detected in different regions of mammalian eyes (16–18), only AQP0 and AQP5 are highly concentrated in the lens (19–21). Subcellular studies have shown that AQP0 is arranged in microdomains of the lens fibers and that the channels have multifunctional properties including cell-to-cell adhesion and water transport (22–27). Mammalian knockout models have further shown that AQP0 is essential for lens development and integrity and that its absence is sufficient to trigger the pathophysiologic condition of cataractogenesis (28). Other studies of mammalian *AQP0* have revealed that a number of mutations in the coding regions of the transmembrane domains (TMDs), the extracellular loop A, or in the intracellular C terminus can disrupt the trafficking of the protein to the plasma membrane resulting in loss of function, lens opacity, and impaired vision or blindness (28–30).

In contrast to mammals, teleosts lack an *AQP5* gene (15), but have been found to retain 2 copies of *AQP0* (*aqp0a* and *-0b*), both of which are highly concentrated in the lens (31–34). However, detectable levels of teleost *aqp0* are found in other tissues, including *aqp0b* mRNA in the ovary (33) and Aqp0a protein in the Sertoli cells of the testis (35). As in mammals, morpholino-based knockdown experiments of *aqp0a* and *-0b* have revealed that both channels are essential for normal lens development and transparency (36, 37), indicating that the major physiologic role of AQP0 is conserved in teleosts.

Functional studies of the duplicated Aqp0a and -0b paralogs have only been conducted for zebrafish (33, 36, 37), with additional measurements of water permeability tested for the Aqp0a channel of the common mummichog (*Fundulus heteroclitus*) (38) and gilthead seabream (*Sparus aurata*) (35). Each study found that, unlike mammalian AQP0, which has a low intrinsic water permeability (39), teleost Aqp0a transports water efficiently when heterologously expressed in *Xenopus laevis* oocytes. By contrast, the heterologous studies on zebrafish Aqp0b have produced conflicting results showing efficient water permeation (33) or dysfunctional channels (36, 37). These latter findings have led to the suggestion that Aqp0b has subfunctionalized and provides functions other than water permeability (12, 36, 37). A separate character of AQP0 channels appears to be an inherent sensitivity to pH and Ca^2+^ (24, 38, 40–42). However, current evidence indicates that the mechanism of pH sensitivity may not be conserved between teleosts and mammals, because the water permeation of common mummichog Aqp0a is reduced by an acidic external pH, whereas the reverse is observed for bovine AQP0 (38, 41). It thus remains to be established whether the alkaline permeation preference of teleost Aqp0a is altered in the Aqp0b paralog or represents an aquatic adaptation compared with the acidic shift of AQP0 in terrestrial mammals.

It is well established that the majority of teleosts retain 2 gene copies arising from a fish-specific whole genome duplication event (R3 WGD) ∼320–350 million years ago (43–47). However, several lineages, including members of the Ostariophysi (48–50) and Protacanthopterygii (51), have experienced an independent R4 WGD. In the case of Salmonidae, this latter event is estimated to have occurred between 88 and 103 million years ago (52, 53). Considering that the average functional lifespan of duplicated non-neofunctionalized genes is <8 million years (54), it is perhaps not surprising that gene fractionation in salmonids is very active (52). Nevertheless, an analysis of 9057 Atlantic salmon (*Salmo salar*) cDNA sequences has provided evidence that many gene duplicates have been retained (55). For the salmonid aquaporin superfamily, this facet has recently been confirmed, with 35 and 42 paralogs, respectively, identified in the genomes of rainbow trout (*Oncorhynchus mykiss*) and Atlantic salmon (15). To date, only 7 of these paralogs have been investigated (56–58), but it is not known whether the Atlantic salmon retains 4 functional aquaporin paralogs in any given subfamily.

In the present study, we therefore researched the Atlantic salmon genome to facilitate isolation and cloning of 4 *aqp0* genes. To address their interrelationships and the prevalence of duplicates in ray-finned fishes, we used Bayesian inference to reconstruct the phylogeny of 78 gnathostome *aqp0* gene products. Subsequently we examined the molecular physiology of the tetraparalogous Aqp0 channels in the Atlantic salmon as a first step toward identifying their potential roles in cataractogenesis, which represents a welfare problem in farmed strains (59–61). We used site-directed mutagenesis to investigate the molecular basis of the pH sensitivity of the Atlantic salmon channels in relation to that of the zebrafish Aqp0a and -0b duplicates. The results suggest that the salmon Aqp0 paralogs may have been retained because of neofunctionalized pH sensitivities.

## MATERIALS AND METHODS

### Animals

Atlantic salmon between 1 and 5 kg of AquaGen origin were held in captivity at the Matredal Aquaculture Research Station (61°N) in Norway. The AquaGen strain is the most common salmon farming strain in Norway and has been under selective breeding for ∼10 generations, originating from a range of wild Norwegian salmon populations collected in the early 1970s (*http://aquagen.no/en/*). Prior to sampling, fish were anesthetized with metomidate (Syndel, Victoria, BC, Canada) and immediately euthanized in accordance with the regulations approved by the governmental Norwegian Animal Research Authority (*http://www.fdu.no/fdu/*). Tissue samples were dissected from 5 fish, immediately frozen in liquid nitrogen, and stored at −80°C for subsequent analyses.

### Cloning of Atlantic salmon *aqp0* cDNAs and genomic sequences

Initial searching of the Atlantic salmon genome database curated at the National Center for Biotechnology Information using the tblastn algorithm and zebrafish Aqp0a and -0b as input identified multiple *aqp0-*bearing contigs from which gene-specific primers were designed. Four nonredundant cDNAs were isolated from the sampled lens tissues by RT-PCR using RNA and a high-fidelity DNA polymerase (EasyA high-fidelity PCR cloning enzyme; Agilent Technologies Santa Clara, CA, USA). Total RNA was purified using the GenElute mammalian total RNA miniprep kit (Sigma-Aldrich, St. Louis, MO, USA), according to the manufacturer’s instructions. cDNA synthesis was performed with 1 *μ*g of total RNA using an oligo dT_(12-18)_ primer (Life Technologies, Carlsbad, CA, USA) and SuperScript II RT enzyme (Life Technologies) as previously described (62). Oligonucleotide primers used to amplify the full-length mRNA sequences of *aqp0a1* and *-0a2* were designed from contigs AGKD01122317 and AGKD01164501 for *aqp0a1* and AGKD01193927 and AGKD01157319 for *aqp0a2*. The PCR conditions were an initial denaturing step for 2 minutes at 94°C, followed by 35 cycles of 94°C for 1 minute, 60°C for 1 minute, and 72°C for 2 minutes, ending with a final elongation at 72°C for 7 minutes. For *aqp0b1* and *-0b2*, partial cDNA sequences bearing the 5′ and 3′ end, respectively, were found on 2 different contigs (AGKD01047775 for *aqp0b1* and AGKD01117189 for *aqp0b2*), which were used for primer design and the cloning of the 3′ and 5′ cDNA ends using 3′ and 5′ RACE kits (Life Technologies). Subsequently, full-length *aqp0b1* and *-0b2* cDNAs were amplified using specific primers as described above. In all cases, the amplified cDNAs were cloned into the pGEM-T Easy vector (Promega, Madison, WI, USA) and sequenced by BigDye Terminator Version 3.1 cycle sequencing on ABI PRISM 377 DNA Analyzer (Applied Biosystems, Foster City, CA, USA).

Genomic sequences containing the full introns and exons of the *aqp0a1*, *-0a2*, *-0b1*, and *-0b2* genes were amplified from genomic DNA by PCR using the same primers and polymerase used for full-length cDNA cloning. The PCR reactions were carried out in the presence of 1.25 M betaine (Sigma-Aldrich), 0.4 *μ*M of each primer, and 100 ng of blood cell-purified DNA, using the EasyA DNA polymerase. The PCR conditions were as follows: denaturing step for 2 minutes at 94°C, followed by 35 cycles of 94°C for 1 minute, 55°C for 1 minute, and 72°C for 4 minutes, ending with a final elongation step at 72°C for 7 minutes. The 5′ flanking regions of *aqp0a1*, *-0a2*, and *-0b1* genes were amplified by PCR using primers designed based on the contig sequences described above. For *aqp0b2*, the primers were designed based on contig AGKD03029857. The DNA fragments were cloned and sequenced as above.

The nucleotide sequences corresponding to the *aqp0* cDNAs and genomic regions were deposited in GenBank with accession numbers KM823661, KM677198–KM677200, and KM876671–KM876680.

### Sequence and phylogenetic analysis

The deduced amino acid sequences of the isolated Atlantic salmon cDNAs were aligned with other gnathostome Aqp0 orthologs retrieved from public sources, including Ensembl, GenBank, National Center for Biotechnology Information whole genome shotgun, transcriptome shotgun assemblies, and expressed sequence tag databases as described previously (15). Alignments were constructed using the multiple sequence alignment based on fast Fourier transform (v7.187) software package (63) and converted to codon alignments using Pal2Nal (64). Molecular phylogenies were inferred using Bayesian (MrBayes, v3.2.2; with 5 million Markov chain Monte Carlo generations) (65) and maximum likelihood (PAUP v4b10-x86-macosx) (66) protocols as described previously (15, 62, 67, 68).

The 3-dimensional structure of bovine AQP0 (2b6p) was obtained from the protein data bank (*rcsb.org*), and *in silico* models of Atlantic salmon Aqp0 channels were built using the model leverage option in the Modeler server (*modbase.compbio.ucsf.edu*), based on the bovine AQP0 template with an ungapped aligned sequence identity of 68–70%. The best scoring models were selected using the slow (Seq-Prf, position–specific iterative-basic local alignment search tool) assignment method, and rendered with MacPymol (*pymol.org*).

### *In silico* analysis of *cis*-acting regulatory sequences in *aqp0* 5′ flanking regions

The 5′ flanking regions of the 4 Atlantic salmon *aqp0* genes were analyzed using TRANSFAC 7.0 Public 2005 software (*http://www.biobase-international.com*) by setting parameters of research to all profiles, using only high-quality matrices and minimizing the sum of both error rates. Only the putative *cis*-acting regulatory sequences corresponding to transcription factor (TF) binding sites showing functional depth score > 90% were selected for the final analysis.

### Real-time quantitative RT-PCR

Total RNA from adult tissues (lens, eye, brain, gills, kidney, middle intestine, rectum, ovary, and testis) was isolated as described above, and first-strand cDNA was synthesized from 0.5 *µ*g total RNA. After 15 minutes of heating at 70°C in the presence of 0.5 *µ*g oligo(dT)_(12-18)_ and 1 mM dNTPs, 40 IU RNase out and 10 IU SuperScript II enzyme were added, and the reaction was completed for 1.5 hours at 42°C. Real-time quantitative RT-PCR amplifications were performed in a final volume of 10 *µ*l with 5 *µ*l Platinum SYBR Green qPCR Supermix-UDG with ROX (Life Technologies), 1 *µ*l of 1:20 diluted cDNA, and 0.5 *µ*M of each specific primer (**Table 1** and Supplemental Fig. S1). The sequences were amplified in duplicate for each sample on 384-well plates using the ABI PRISM 7900HT sequence detection system (Applied Biosystems). The amplification protocol was an initial denaturation and activation step at 50°C for 2 minutes and 5°C for 10 minutes, followed by 40 cycles of 95°C for 15 seconds and 63°C for 1 minute. After the amplification phase, a temperature-determining dissociation step was carried out at 95°C for 15 seconds, 60°C for 15 seconds, and 95°C for 15 seconds. For normalization of cDNA loading, all samples were run in parallel using the 18s ribosomal protein (*rps18*) as a reference gene, because its expression between experimental samples did not show significant differences (data not shown). To estimate the primer efficiencies, a standard curve was generated for each primer pair from 10-fold serial dilutions (from 1 to 0.00001) of a pool of mixed lens cDNA templates. Standard curves represented the cycle threshold value as a function of the logarithm of the number of copies generated, defined arbitrarily as 1 copy for the nondiluted standard. All calibration curves exhibited correlation coefficients >0.99, and the corresponding quantitative RT-PCR efficiencies ranged from 1.9 to 2.0.

**TABLE 1.** Primer pair sequences, amplicon size, annealing temperature, and efficiency for Atlantic salmon aqp0 and reference gene used in quantitative RT-PCR

Gene	GenBank accession no.	Forward/reverse (5′ to 3′ end)	Amplicon (bp)	Annealing temperature (°C)	Primer efficiency*^a^*
*aqp0a1*	KM823661	TCAACCCTACCCAACACACA/	109	60	2.05
		TGAGGAGGGTGAGAAAGGTG			
*aqp0a2*	KM677198	CCACTGACCCTTACCCATACC/	128	60	2.04
		CAGGAGTGACCCATTCCCTA			
*aqp0b1*	KM677199	TTGATGTTTGACTGCCTTCG/	112	60	2.03
		AGCCAATCAGTTGGAAGACAA			
*aqp0b2*	KM677200	AGCTAGCTGACTGCCAAAGG/	142	60	1.93
		CGGAAAATGTTCTTGGGAAA			
*rps18*	AJ427629	TACAGTGAAACTGCGAATGG/	153	60	1.99
		GCATGGGTTTTGGGTCTG			

a Efficiency was determined from standard curves generated from 10-fold serial dilutions of first-stranded cDNA template from lens. In all cases, the correlation coefficients were 0.99.

### Functional characterization of Aqp0 paralogs in *X. laevis* oocytes

Constructs for heterologous expression in *X. laevis* oocytes were generated by subcloning full-length *aqp0* cDNAs from Atlantic salmon and zebrafish (GenBank accession numbers NM_001003534 and NM_001020520) into the pT7Ts expression vector (69) by introducing *Eco*RV and *Spe*I sites at the 5′ and 3′end, respectively. Point mutations in the sequences were introduced using the Quickchange site-directed mutagenesis kit (Stratagene; Agilent Technologies). All constructs in pT7Ts were resequenced as above to assure that the correct mutations were present. Isolation of stage V–VI oocytes and cRNA synthesis were carried out as previously described (69, 70). Oocytes were transferred to modified Barth’s solution [MBS; 88 mM NaCl, 1 mM KCl, 2.4 mM NaHCO_3_, 0.82 mM MgSO_4_, 0.33 mM Ca(NO_3_)_2_, 0.41 mM CaCl_2_, 10 mM 4-(2-hydroxyethyl)-1-piperazineethanesulfonic acid, and 25 *μ*g/ml gentamycin, pH 7.5] and injected with 50 nl of distilled water (negative control) or 50 nl of water solution containing 10 ng of cRNA. Two days after injection, the oocytes were transferred to isotonic MBS (200 mOsm) at pH 6, 7.5, or 8.5 for 15 minutes and then transferred to 10-fold diluted MBS (20 mOsm) at the same pH. Oocyte swelling and determination of the osmotic water permeability (*P*_f_) were calculated as previously described (70) using an estimated surface area of 9× the geometric area. Each experiment was carried out ≥3 times on separate oocyte batches.

### Statistics

Data are presented as the mean ± sem. Data analyses were carried out by 1- or 2-way ANOVA, after log-transformation of the data when needed, followed by the Duncan multiple range test. A value of *P* < 0.05 was considered significant.

## RESULTS

### Structure and genomic organization of Atlantic salmon *aqp0* genes

**Figure 1.**
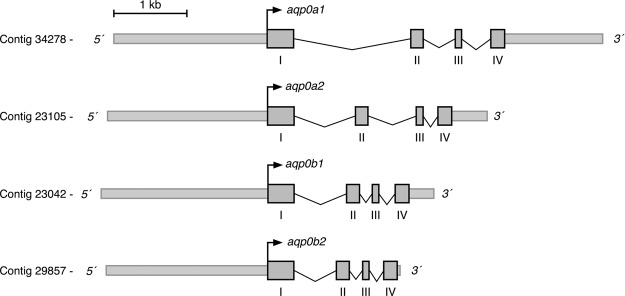
Genomic organization of Atlantic salmon *aqp0* genes. Schematic representation of *aqp0a1*, *-0a2*, *-0b1*, and *-0b2* gene loci. Gray boxes indicate exons with coding regions only.

Initial searching of the Atlantic salmon genome identified 4 paralogous *aqp0* genes, which, following molecular phylogenetic characterization (see below), were termed *aqp0a1*, *-0a2*, *-0b1*, and *-0b2* (**Fig. 1**) The genomic sequences were used to design paralog-specific primers to amplify the full-length cDNAs from total RNA isolated from lens tissues, as well as the complete genes from genomic DNA. These experiments revealed that the 4 Atlantic salmon *aqp0* genes were each split into 4 exons of conserved lengths (360, 165, 84, and 183 bp for exons 1, 2, 3, and 4, respectively), whereas the size of the intronic regions were more divergent (581–1618, 180–661, and 205–406 bp for introns 1, 2, and 3, respectively) (Fig. 1). Compared with the *aqp0a1* mRNA, nucleotide identity varied between 83–85% for the *aqp0b* paralogs and 94% for the *aqp0a2* paralog.

A region of ∼2.2 kb corresponding to the 5′ flanking region of each *aqp0* gene was further amplified by PCR. For *aqp0a1* and *-0b1*, however, 2 different variants of the 5′ flanking region, with a deletion of ∼200 bp, were found. The 4 salmon *aqp0* paralogs each encode protein products of 263 amino acids, showing 6 TMDs and 2 Asn-Pro-Ala (NPA) motifs, typical for members of the aquaporin superfamily. The amino acid identities reflected the mRNAs with 84–87% between the Aqp0a and -0b types and 96% between the Aqp0a1 and -0a2, and Aqp0b1 and -0b2 R4 duplicates.

### Phylogenetic relationships

**Figure 2.**
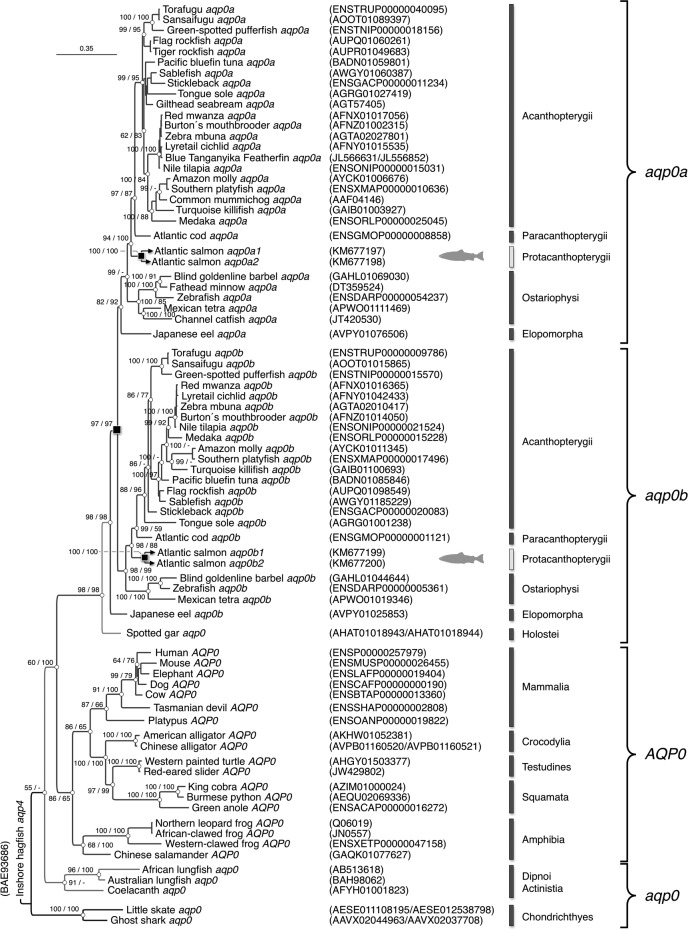
Phylogenetic relationships of Aqp0 in Gnathostomata. Bayesian majority rule consensus tree of a codon alignment of vertebrate *aqp0* orthologs resulting from 5 million Markov chain Monte Carlo generations and a burn-in of 25%. Posterior probabilities of the codon/amino acid analyses are shown at each node, where - indicates <50%. The tree is rooted with Inshore hagfish (*Eptatretus burgeri*) *aqp4*, with the scale bar indicating the rate of nucleotide substitution per site. Whole genome duplication events are shown as black squares at the relevant nodes.

Bayesian inference of 78 gnathostome *aqp0* codon and deduced amino acid sequences revealed that Chondrichthyes, Sarcopterygii, and actinopteryigian Holostei harbor single genes, whereas the majority of Teleostei retain 2 *aqp0* paralogs (*aqp0a* and *-0b*). Although only single paralogs are shown for some teleost species, such as common mummichog *aqp0a*, it is not yet possible to determine whether the absence of *aqp0b* represents gene loss, because of the lack of an available genome. The high posterior probability (97%) separating the teleost *aqp0a* and *-0b* clusters is thus consistent with R3 WGD at the root of the crown clade (**Fig. 2**). The 4 Atlantic salmon paralogs (*aqp0a1*, *-0a2*, *-0b1*, and *-0b2*), clustered as duplicated members of the Protacanthopterygii within the respective teleost *aqp0a* and *-0b* subfamilies. Comparison of the 4 *aqp0* paralogs assembled from the rainbow trout genome revealed that the interspecific amino acid identities between each of the Aqp0a1, -0a2, -0b1, and -0b2 orthologs were 98.1%, 99.6%, 98.9%, and 99.2%, respectively, and thus consistent with an extra tetraploidization in Salmonidae.

### The 5′ flanking regions of Atlantic salmon *aqp0* genes contain high numbers of putative sites for lens development-related transcription factors

The 2.2-kb 5′ flanking genomic sequence of the 4 Atlantic salmon *aqp0* genes were analyzed for putative *cis*-acting regulatory sequences using the TRANSFAC 7.0 software. These analyses revealed the presence of consensus sequences for core promoter elements important for the interaction with the basal transcription machinery, such as several TATA boxes and Sp1 and AP1. The 4 *aqp0* 5′ flanking sequences also contained a high number (>100) of putative binding sites for 13 transcription factors, including musculoaponeurotic oncogene homolog (MAF), myeloid ecotropic viral integration site (MEIS), paired-like homeodomain 3 (PITX3), or sex determining region Y-box (SOX), that are known to be involved in ectodermal signaling for lens differentiation, proliferation, and survival (**Table 2** and Supplemental Table S1). A lower number (<15) of sites for factors related to retina development, such as Optix or VSX2, were also found in the 4 genes, with *aqp0b1* harboring the majority of these factors (Supplemental Table S1). Interestingly, the sequences potentially involved in lens development were more conserved among the 4 *aqp0* paralogs than those related to eye development (Table 2; Supplemental Table S1). Another, 42 different putative regulatory elements, including DMBX1, NEUROD1, and NEUROG2, that are specifically involved in brain and central nervous system development were identified among the *aqp0* genes, with the *aqp0b1* 5′ flanking region showing the highest number of different potential sites (38) compared with the other *aqp0* genes (11, 24, and 22 in *aqp0a1*, *-0a2*, and *-0b2*, respectively; Supplemental Table S1). The flanking region of the *aqp0a1*, *-0a2*, *-0b1*, and *-0b2* also contained 10, 14, 13, and 18 different consensus sites, respectively, for TFs associated with gonad development, including doublesex and mab-3 related transcription factor 1 (DMRT1), Wilms tumor 1 (WT1), or AR for testicular differentiation and FOXL2, FIGLA, or ER for ovarian development (Table 2; Supplemental Table S1). In addition, a variable number of putative binding sites for other tissue-specific transcription factors, such as in the muscle or kidney, were differentially found in the promoter regions of the 4 Atlantic salmon *aqp0s* (Supplemental Table S1).

**TABLE 2.** Potential binding sites of TFs relevant during lens and gonad development identified in the 5′ flanking regions of the Atlantic salmon aqp0a1, -0a2, -0b1, and -0b2 genes

Process	TF symbol	No. sites in 5′ flanking region				Function
		*aqp0a1*	*aqp0a2*	*aqp0b1*	*aqp0b2*	
Lens development	GATA3	20	19	19	9	Lens cells proliferation and differentiation
	MAF	11	16	18	17	Lens fiber differentiation
	MAFB	25	30	26	34	Heterologous expression of crystallins and MIP
	MEIS	13	20	22	12	Lens ectoderm specification
	PAX6	16	23	25	18	Lens placode formation/specification
	PITX3	3	3	4	6	Lens cells proliferation, differentiation and survival
	SOX	17	15	25	13	Lens fiber, vesicle and placode formation and differentiation
	SP3	2	10	7	11	Regulation of MIP gene expression in lens
Gonad development	DMRT1	1	0	0	0	Sertoli cell and germ cell development
	ETV5	1	2	0	1	Spermatogonial stem cell self-renewal
	MYBL1	0	1	1	3	Male germ cell meiosis
	NR6A1	0	1	0	1	Spermatid nuclear elongation and condensation
	WT1	0	5	9	12	Sertoli cell development
	AR	6	20	8	11	Gonad development and function
	ER	5	10	5	6	Ovulatory function, germ cell development

See Supplemental Table S1 for the complete list of potential TF binding sites with a score >0.9 identified with the TRANSFAC 7.0 software and references for the inferred function.

### Atlantic salmon *aqp0* paralogs are highly, but not exclusively, expressed in the lens

**Figure 3.**
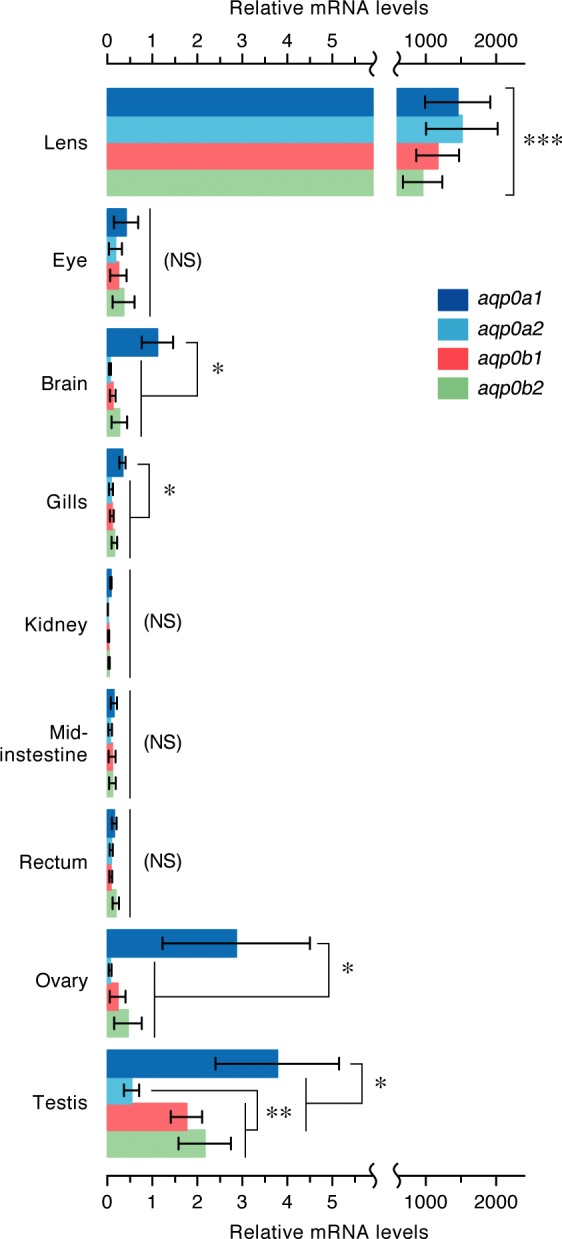
Tissue expression pattern of Atlantic salmon *aqp0* genes. Tissue distribution of *aqp0a1*, *-0a2*, *-0b1*, and *-0b2* transcripts determined by quantitative RT-PCR and using *rps18* as reference gene. Data are means ± sem (*n* = 5 fish). Significant differences (*P < 0.05; **P < 0.01; ***P < 0.001) between paralogs in each tissue are indicated. The bracket indicates significant differences of the expression levels in the lens with respect the other tissues. NS, not significant.

To design paralog-specific oligonucleotide primers for quantitative RT-PCR experiments, the 3′ UTR of the *aqp0a1*, *-0a2*, and *-0b1* mRNAs were amplified by 3′ rapid amplification of cDNA ends. Because of the high sequence similarity of the fourth exon of *aqp0b1* and *-0b2* (97.3% identity), the 3′UTR of *aqp0b2* could not be specifically amplified, and the 5′ UTR was used instead. The 5′ and 3′ terminal sequences were then aligned and specific primers with similar efficiencies designed for each paralog (Table 1). Quantitative RT-PCR analyses of the pattern of mRNA expression in different adult tissues subsequently revealed that the 4 *aqp0s* transcripts were highly concentrated in the lens, with no significant difference between the paralog titers (**Fig. 3**). However, *aqp0a1, -0a2, -0b1,* and *-0b2* mRNAs could also be detected in the delensed eye, brain, gills, kidney, mid intestine, rectum, ovary, and testis, although at much lower expression levels (∼1000-fold less) than in the lens (Fig. 3). Compared with the other transcripts, *aqp0a1* was more concentrated in the brain, gills, and ovary, whereas in testis, the expression levels of all 4 mRNAs were elevated, with *aqp0a1* showing the highest levels (Fig. 3). These data confirmed that, although the mRNA levels in the lens are greatly concentrated with respect to the other tissues, *aqp0* expression in Atlantic salmon and other species of teleost (33, 35) is not lens specific.

### Atlantic salmon Aqp0 paralogs retain unique pH sensitivities

**Figure 4.**
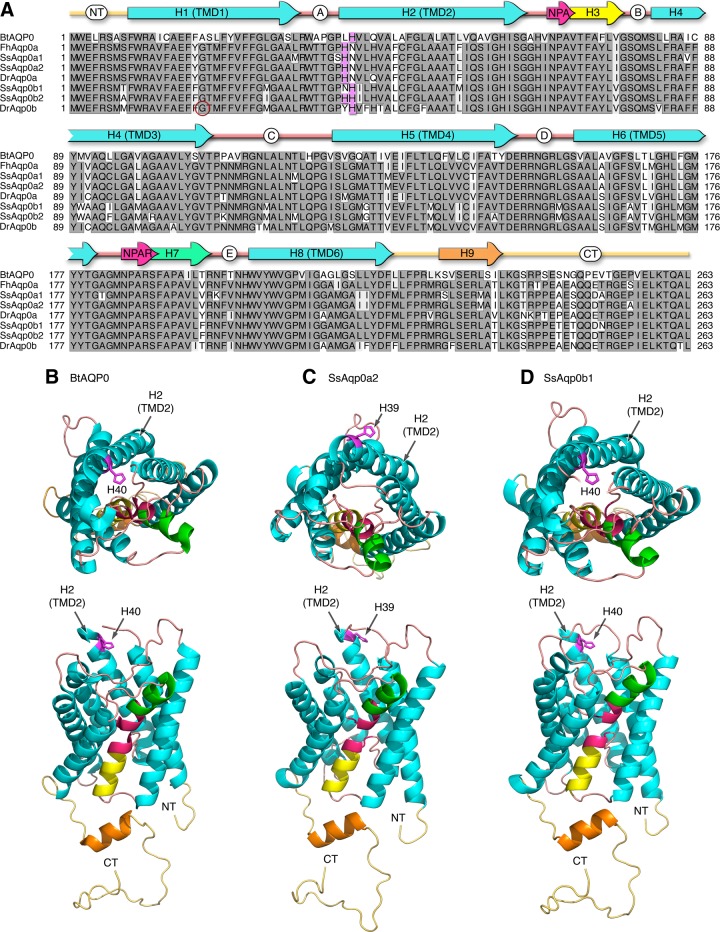
Structural analysis of tetrapod and teleost AQP0. *A*) Amino acid alignment of Atlantic salmon and representative teleost Aqp0 sequences in relation to bovine AQP0. Fully conserved residues are boxed in dark gray, whereas the His residue involved in pH sensitivity is shaded in magenta. The zebrafish allelic variant at position Gly^19^ is circled in red. Conserved Asn-Pro-Ala (NPA) motifs (pale red arrows) are highlighted with *α*-helical regions shown for TMDs 1–6 (light blue arrows), hemihelices 3 (yellow arrow), 7 (green arrow), and 9 (orange arrow), intra- and extracellular loops (A–E; pink lines) with the N (NT) and C (CT) termini (palid orange lines). Taxa as follows: Bt, *Bos taurus*; Fh, *Fundulus heteroclitus*; Ss, *Salmo salar*; Dr, *Danio rerio*. *B–D*) Extracellular (upper) and lateral (lower) views of bovine AQP0 (*B*) and Atlantic salmon Aqp0a2 (*C*) and Aqp0b1 (*D*) are rendered with MacPymol.

The amino acid alignment of the 78 gnathostome Aqp0 orthologs revealed that His^40^ at the beginning of TMD2 close to loop A, which is known to affect the pH sensitivity of the water channel activity of bovine AQP0 (40, 41), is conserved in Eutheria, Metatheria, and the teleost b-type Aqp0s. However, it is not found in Chondrichthyes, Actinistia (coelacanths), Dipnoi (lungfishes), Amphibia, Sauropsida (reptiles and birds), Prototheria (platypus), Holostei (spotted gar), or the teleost a-type aquaporins. In all cases, an Asn^40^ is encoded instead of the His^40^, whereas in the teleost a-type Aqp0s, a His is encoded at position 39. This latter His^39^ is also prevalent in the b-type water channels of Acanthomorpha (spiny ray-finned teleosts). Interestingly, the Atlantic salmon displays the a-type His^39^ in both of the Aqp0a1 and -0a2 paralogs, but with a nonconserved Ser encoded 1 amino acid upstream in the Aqp0a1 paralog. Conversely the salmon Aqp0b1 paralog displays the conserved His^40^, whereas the Aqp0b2 encodes both His^39^ and His^40^ as found in the majority of Acanthomorpha (**Fig. 4*A***). Based on the crystal structure of bovine AQP0 (Fig. 4*B*) (71), *in silico* modeling of Aqp0a2 and -0b1 (Fig. 4*C, D*) revealed that the His^40^ residues of bovine AQP0 and Atlantic salmon Aqp0b1 face the inner vestibular opening of the water pore, whereas His^39^ in Aqp0a2 lies outside of the pore.

**Figure 5.**
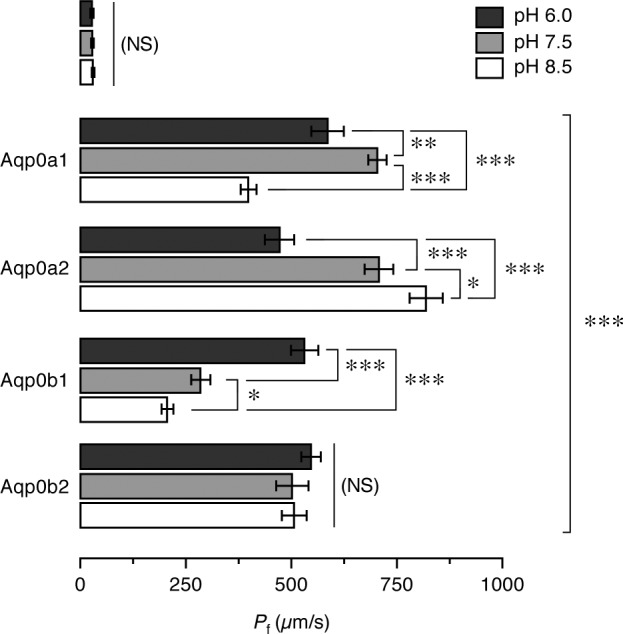
Functional characterization of Atlantic salmon Aqp0 paralogs. Osmotic water permeability (*P*_f_) of *X. laevis* oocytes injected with water (W) or cRNA encoding Aqp0a1, -0a2, -0b1, and -0b2 and exposed to different pHs before and during the swelling assays. The *P*_f_ was calculated using an estimated surface area of 9× the geometric area. Data are the mean ± sem (*n* = 12 oocytes per treatment) of 3 independent experiments. Significant differences (*P < 0.05; **P < 0.01; ***P < 0.001) for each aquaporin at the 3 pHs are indicated. The bracket indicates significant differences with respect water-injected oocytes. NS, not significant.

The functional properties of the 4 salmon Aqp0 paralogs were investigated using *X. laevis* oocytes as a heterologous expression system. Oocytes expressing Aqp0a1, -0a2, -0b1, or -0b2 showed a 20- to 25-fold increase in *P*_f_ with respect to water-injected (control) oocytes (**Fig. 5**). However, each paralog displayed a different pH sensitivity (Fig. 5). Aqp0a1 oocytes were more permeable at external neutral pH (7.5) and showed lower permeability at acidic and alkaline pH (6.0 and 8.5), whereas Aqp0a2 oocytes showed a progressive increase of water transport associated with an increase in pH, with the highest *P*_f_ at alkaline pH. In contrast, the *P*_f_ of oocytes expressing Aqp0b1 increased under acidic conditions (pH 6.0), whereas that of Aqp0b2 oocytes was not affected by changes in the external pH. These data therefore indicated a unique pH regulation for each of the 4 Atlantic salmon Aqp0 paralogs.

### Alkaline and acidic pH sensitivities of salmon Aqp0a2 and -0b1 are conserved in zebrafish Aqp0 channels

**Figure 6.**
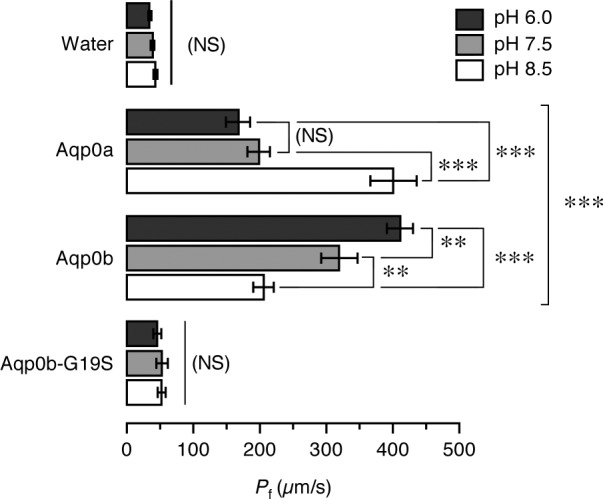
Both zebrafish *aqp0a* and *-0b* paralogs encode functional water channels. Osmotic water permeability (*P*_f_) of *X. laevis* oocytes injected with water or cRNA encoding zebrafish wild-type Aqp0a or -0b or the Aqp0b-G19S mutant. The *P*_f_ was calculated using an estimated surface area of 9× the geometric area. Oocytes were exposed to different pH conditions before and during the swelling assays. Data are the mean ± sem (*n* = 8–12 oocytes per treatment) of 4 independent experiments. Significant differences (*P < 0.05; **P < 0.01; ***P < 0.001) for each construct at the 3 pHs are indicated. The bracket indicates significant differences with respect water-injected oocytes, NS, not significant.

To determine which of the pH sensitivities observed in the salmon Aqp0 paralogs could be potentially conserved in other teleosts, we reexamined the water permeation properties of the duplicated zebrafish Aqp0a and -0b paralogs. Two different forms of zebrafish Aqp0b have been reported, which differ in a Gly or Ser at position 19 in the N terminus (accession numbers BC098535 and NM_001020520, respectively; Fig. 4*A*). The form bearing Gly^19^, isolated in our laboratory, is conserved in other teleosts, and functional when expressed in *X. leavis* oocytes (33), whereas the form with a Ser^19^ residue has been reported to lack water transport (36, 37). To confirm that this single nucleotide polymorphism can alter the functional properties of zebrafish Aqp0b, a zebrafish Aqp0b-G19S mutant was constructed by site-directed mutagenesis and tested in oocytes exposed to different pH. The results of these experiments showed that zebrafish Aqp0a and -0b display opposite pH sensitivities, with the highest *P*_f_ elicited by Aqp0a expressing oocytes at alkaline pH, whereas that of the Aqp0b expressing oocytes was at acidic pH, thus closely reflecting the pH permeation properties observed for the Atlantic salmon Aqp0a2 and -0b1 orthologs (**Fig. 6**). As expected, the water permeability of oocytes expressing zebrafish Aqp0b-G19S was completely abolished (Fig. 6), confirming that zebrafish may harbor a nonfunctional Aqp0b allele (36, 37).

### Single His at the entrance of the water pore regulates pH sensitivity

**Figure 7.**
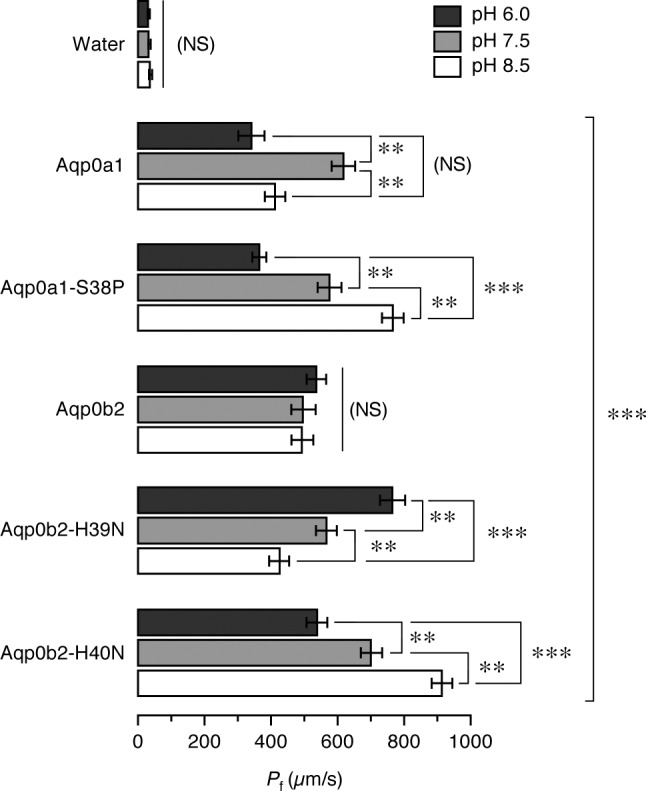
Role of Ser^38^ and His^39^-His^40^ on Atlantic salmon Aqp0a1 and -0b2 pH sensitivity. Osmotic water permeability (*P*_f_) of *X. laevis* oocytes injected with water or cRNA encoding wild-type Aqp0a1 or Aqp0b2 or different mutants in which Ser^39^ was replaced by Pro in Aqp0a1 (Aqp0a1-S39P) or in which a single His in position 39 or 40 replaced the double His in Aqp0b2 (Aqp0b2–H40N and Aqp0b2-H39N, respectively). The *P*_f_ was calculated using an estimated surface area of 9× the geometric area. Oocytes were exposed to different pH before and during the swelling assays. Data are the mean ± sem (*n* = 12 oocytes per construct) of a representative experiment. Significant differences (*P < 0.05; **P < 0.01; ***P < 0.001) for each construct at the 3 pHs are indicated. The bracket indicates significant differences with respect water-injected oocytes. NS, not significant.

The preceding experiments, together with previously published data (38, 40, 41), indicated that teleost Aqp0 a-type with His at position 39, such as Atlantic salmon Aqp0a2, zebrafish Aqp0a, and killifish Aqp0a, show maximum water channel activity at alkaline pH, whereas teleost Aqp0 b-type, such as Atlantic salmon Aqp0b1, zebrafish Aqp0b, and bovine AQP0, all bearing the His^40^ residue, are more permeable at acidic pH. However, salmon Aqp0a1 showed the highest permeability at neutral pH, despite a His residue at position 39. Reinspection of the amino acid alignment revealed that the nonconserved Ser^38^ of Aqp0a1 is usually represented by a conserved Pro at position 38 (Fig. 4*A*). Mutation of the Ser^38^ into a Pro in Aqp0a1 (Aqp0a1-S38P) recovered maximum permeability at alkaline pH, thus converting the channel into a bona fide a-type Aqp0 (**Fig. 7**). Similarly, when one of the His^39^ and His^40^ residues of Atlantic salmon Aqp0b2, which is not affected by pH, was replaced by an Asn (Aqp0b2-H39N or Aqp0b2-H40N), the channel became more permeable at acidic or basic pH, respectively (Fig. 7). These data therefore demonstrate that the pH sensitivity of the Atlantic salmon Aqp0 paralogs is primarily determined by the relative position of a single His in the second transmembrane domain close to loop A facing the entrance of the pore.

## DISCUSSION

It has recently been reported that almost half of the duplicated protein-coding genes arising from the R4 WGD event in Salmonidae are lost in extant species (52). In the trout genome, the cluster of genes with retained duplicates, which neofunctionalized, subfunctionalized, or acquired different expression patterns, is mostly related to visual perception (52). The present study in Atlantic salmon supports these findings because we found that tetraparalogous *aqp0* genes encode functional water channels that are equally expressed in the ocular lens at high titers, but with unique pH sensitivities.

The high expression of Atlantic salmon *aqp0a1*, *-0a2*, *-0b1*, and *-0b2* in the lens agrees with the finding that the 5′ flanking regions of each of the 4 genes bear elevated numbers of putative binding sites for TFs important for lens development and differentiation in mammals, including PITX3 (72, 73), SOX and MEIS (74), or avian-MAF (75). Such equally abundant expression of *aqp0* paralogs in the salmon lens is intriguing because it implies that each has been positively selected for nonredundant functions. In zebrafish it has been established that both Aqp0a and -0b are necessary for lens development and transparency with the water permeability mediated by Aqp0a being essential; however, the Aqp0b paralog may play additional roles not related to water transport (12, 37). Therefore, in Atlantic salmon, it is possible that each *aqp0* paralog plays distinct but complementary functions in the lens, although this hypothesis needs further investigation.

Despite the high expression titers of *aqp0* genes in the Atlantic salmon lens, *aqp0a1*, *-0a2*, *-0b1,* and *-0b2* transcripts were also detected in other tissues, particularly in the gonads, as previously document for other teleosts (33, 35). These observations confirm that *aqp0* in Teleostei is not lens specific. The extraocular *aqp0* expression in salmon is supported by the presence of putative sites for TFs related to gonad development, and in particular Sertoli cell function in the testis, such as ETV5 (76), androgen receptor (77, 78), DMRT1 (79), or WT1 (80), in the 5′ flanking region of the *aqp0* paralogs. Although the role of Aqp0 in the gonads is unknown, this channel has been localized in Sertoli cells of gilthead seabream (35), and the rat (81), where it might regulate the secretion of intratubular fluid in addition to reinforcing Sertoli cell junctions (82).

Our finding that the tetraploid Atlantic salmon retains 4 functional Aqp0 water channels derived from the R4 WGD event in Salmonidae indicates that Aqp0 paralogs have been positively selected in this lineage for ∼100 million years. The additional confirmation that both zebrafish Aqp0a and -0b paralogs are functional water transporters when heterologously expressed in *X. laevis* oocytes (33) further suggests that such positive selection has existed since the R3 WGD event, and thus for ∼350 million years. These findings contrast another study, however, which reported that zebrafish Aqp0b is not a functional water channel (36). As we have shown in this work, this apparent discrepancy is caused by the use in the latter study of an alternative allele of zebrafish *aqp0b*, which encodes a point mutation of Ser^19^ instead of the conserved Gly^19^ in the N terminus of the protein. The Ser^19^ mutation completely abolishes Aqp0b-mediated water channel activity. Nevertheless, although we show that water transport is most likely an inherent property of zebrafish Aqp0b, a previous study showed that an impermeable mutant of common mummichog Aqp0a (MIPfunN68Q) could not rescue morpholino knockdown of zebrafish Aqp0a but was able to rescue the phenotype induced by an Aqp0b morpholino (37), suggesting that water permeability might not be required for Aqp0b function in the zebrafish lens.

Our data further demonstrate that teleost a-type Aqp0 water channels display maximum water permeability at alkaline pH, whereas some of the b-type channels are more permeated at acidic pH as found for bovine AQP0. This latter acidic permeation preference is therefore likely conserved in metatherian and eutherian mammals because of their retention of His^40^. In Atlantic salmon, however, Aqp0a1 is more permeable at neutral pH, whereas Aqp0b2 does not display pH sensitivity. Previous studies on bovine and common mummichog AQP0 suggested that external His residues in loops A and C that span the outer vestibule of the channel contribute to pH sensitivity (41). In the present work, site-directed mutagenesis experiments show that the differential pH regulation of Atlantic salmon Aqp0 paralogs is determined by the position of a single His located at the entrance of the water pore. However, the sensitivity of Aqp0a1 at neutral pH can be shifted to alkaline pH by the S38P mutation, suggesting that the effect of His^39^ protonation on the permeability of the pore is also modulated by the microenvironment surrounding this residue. Considering that the p*K*_a_ of the His imidazole group is 6.0, we speculate that charge may play a gating role in Aqp0b1 at pH values > 6, whereas protonation of His^40^ at acidic pH would not only alleviate the negative electrostatic gating, but may move His^40^ away from the central pore because of additional Van der Waals forces, thus facilitating water transport. Conversely, protonation of His^39^, which lies on the outside of Aqp0a2 TMD2, could have the opposite effect at acidic pH, which together with an allosteric realignment of loop A may partially occlude the pore entrance. The significance of the S38P substitution in Aqp0a1 is likely associated with the conformational unwinding of the *α*-helical terminus of TMD2 and thus the positioning of His^40^ over the pore entrance in a-type channels, thus altering pH sensitivity, whereas the opposing arrangement of His^39^ and His^40^ present in Aqp0b2 cancel each other’s effect, leaving this type of channel insensitive to pH modulation.

The physiologic implications of the different pH sensitivities of Atlantic salmon Aqp0 paralogs remain intriguing. It seems reasonable to suggest that the retention of the tetraparalogous salmon Aqp0 channels may be caused by positive selection associated with the neofunctionalization of pH sensitivities to encompass a broad range of aquatic conditions. For example, the paralog-specific pH sensitivities may provide compensatory water transport between the lens fibers when juveniles migrate from acidic freshwater to alkaline seawater during smoltification or when adults reenter freshwater from their oceanic sojourns during the spawning migration. It is also known that the pH of the lens varies from the superficial layers to the nuclear fibers (83, 84). However, the potential differential localization of the salmon Aqp0 paralogs in the lens fiber cells remains to be investigated. In addition, it has been reported that cell-to-cell junctional strength is dependent on acid-base homeostasis (85). The reconstitution of AQP0 into large unilamellar liposomes promotes a fast liposome aggregation at acid pH (86), which is the pH at which the permeability of the channel is the highest. Therefore, the potential role of pH in regulating the water transport and adhesive function of each salmon Aqp0 paralog should be investigated in the future.

In summary, the present study shows that, in contrast to the single *aqp0* genes in Chondrichthyes, Sarcopterygii, Holostei, and the duplicated Aqp0a and -0b paralogs found in the majority of Teleostei, Atlantic salmon retains 4 *aqp0* genes that encode functional water channels with paralog-specific pH sensitivities. Site-directed mutagenic experiments demonstrate that the position of a histidine at the extracellular end of TMD2 is critical for pH-regulated water permeability. Comparison of the salmon Aqp0 permeation properties with the zebrafish orthologs confirm that both zebrafish Aqp0a and -0b are functional water transporters and that the zebrafish channels, respectively, display the alkaline and acidic sensitivities determined for the salmon Aqp0a2 and -0b2 paralogs. Based on the position of the histidine in the amino acid alignment of 78 gnathostome Aqp0 proteins, the present findings suggest that the alkaline pH control of water permeation is conserved in teleost a-type channels, whereas the acidic pH sensitivity regulated by His^40^ in some teleost b-type channels is conserved in mammals but not in other Tetrapoda, basal Sarcopterygii, Holostei, or Chondrichthyes.

## Supplementary Material

Supplemental Data

## Abbreviations

AQP: aquaporin
MS: modified Barth’s solution
TF: transcription factor
TMD: transmembrane domain
WGD: whole genome duplication
