# Supplementary material for: The pH sensitivity of Aqp0 channels in tetraploid and diploid teleosts
Source: FASEB J. 2015 Feb 9;29(5):2172–84. doi: 10.1096/fj.14-267625 (PMC4423293; doi:10.1096/fj.14-267625)
Supplement: Supplemental Data [file supp_29_5_2172__index.html]

The pH sensitivity of Aqp0 channels in tetraploid and diploid teleosts — The pH sensitivity of Aqp0 channels in tetraploid and diploid teleosts — Supplemental Data 

# The pH sensitivity of Aqp0 channels in tetraploid and diploid teleosts

## Supplemental Data

**Files in this Data Supplement:**

- Supplemental Data
- Supplemental Data
- Supplemental Data
