# Supplementary material for: The pH sensitivity of Aqp0 channels in tetraploid and diploid teleosts
Source: FASEB J. 2015 Feb 9;29(5):2172–84. doi: 10.1096/fj.14-267625 (PMC4423293; doi:10.1096/fj.14-267625)
Supplement: Supplemental Data [file supp_fj.14-267625_Supplemental_Table1.pdf]

			No. Binding sites						
**Group**	**TF symbol**	**Description**	***aqp0a1***	***aqp0a2***	***aqp0b1***	***aqp0b2***	**Tissue**	**Functions**	**References**
**Lens development**	GATA3	GATA binding protein 3	20	19	19	9	Lens	Lens cells proliferation and differentiation	Maeda et al., 2009, Dev Dyn. 238(9):2280-91
	MAF	V-maf avian musculoaponeurotic fibrosarcoma oncogene homolog	11	16	18	17	Lens	Lens fiber differentiation	Reza et al., 2007. Differentiation,75(8):737-44; Yoshida et al., 2002, Genes Cells.7(7):693-706
	MAFB	V-maf avian musculoaponeurotic fibrosarcoma oncogene homolog B	25	30	26	34	Lens	Ectopic expression of crystallins and MIP	Ogino et al., 2012, Dev Biol. 363(2):333-47
	MEIS1	Meis homeobox 1	7	12	12	6	Lens	Lens ectoderm specification	Ogino et al., 2012, Dev Biol. 363(2):333-47; Zhang et al., 2002, Genes Dev., 16, 2097-2107.
	MEIS2	Meis homeobox 2	6	8	10	6	Lens	Lens ectoderm specification	Ogino et al., 2012, Dev Biol. 363(2):333-47; Zhang et al., 2002, Genes Dev., 16, 2097-2107.
	PAX6	Paired box 6	16	23	25	18	Lens	Lens placode formation/specification	Ogino et al., 2012, Dev Biol. 363(2):333-47
	PITX3	Paired-like homeodomain 3	3	3	4	6	Lens	Lens cells proliferation, differentiation and survival	Medina-Martinez et al., 2009, Dev Dyn. 238(9):2193-201.; Shi et al., 2005, Mech Dev. 122(4):513-27; Ahmad et al., 2013, Int J Dev Biol. 2013;
	SOX1	SRY (sex determining region Y)-box 1	11	7	14	7	Lens	Lens fiber differentiation	Ogino et al., 2012, Dev Biol. 363(2):333-47
	SOX2	SRY (sex determining region Y)-box 2	6	7	9	6	Lens	Lens vesicle formation/specification	Ogino et al., 2012, Dev Biol. 363(2):333-47
	SOX3	SRY (sex determining region Y)-box 3	0	1	2	0	Lens	Lens placode formation	Köster et al., 2000, Mech Dev. 95(1-2):175-87.
	SP3	Sp3 transcription factor	2	10	7	11	Lens	MIP gene expression regulation	Kim et al., 1999, Mol Vis. Jul 15;5:12
	TFAP2A	Transcription factor AP-2 alpha	4	5	6	4	Lens	Epithelial cell polarity and lens cell adhesion	West-Mays et al., 1999, Dev. Biol. 206(1):46-62.; Makhani et al., 2007, Mol Vis. 13:1215-25.; Kerr et al., 2014, Dev Dyn. doi: 10.1002/dvdy.24
	V-Maf	Avian musculoaponeurotic fibrosarcoma (v-maf) AS42 oncogene homolog	1	2	3	3	Lens	Embryonic lens fiber development	Narumi et al., 2014, Am J Med Genet A. 164A(5):1272-6.
	**TOTAL**		**112**	**143**	**155**	**127**			
**Eye development**	BHLHE22	Basic helix-loop-helix family, member e22	0	0	2	0	Retina	Retinal cone cells pecification	Feng et al., 2006, Development. 133(24):4815-25.
	FOXN4	Forkhead box N4	1	0	0	0	Retina	Retinal neural progenitor differentiation	Islam et al., _2013, Biol Open.2(11):1125-36
	LMX1B	LIM homeobox transcription factor 1, beta	2	1	1	0	Cornea	Anterior segment development	Pressman et al., 2000, Genesis. 26(1):15-25
	Optix	CG18455 gene product from transcript CG18455-RC	5	5	5	5	Retina	Retinal patterning and determination network	Li et al., 2013, Dev Biol. 381(1):50-61
	OTX2	Orthodenticle homeobox 2	0	0	1	3	Retina	Retinal pigment epithelium specification	Beby and Lamonerie, 2013, Exp Eye Res. 111:9-16.
	RAX	Retina and anterior neural fold homeobox	2	1	2	0	Retina	Retinal progenitor proliferation and cell fate	Muranishi et al., _2012, Dev Growth Differ. 54(3):341-8.
	RORB	RAR-related orphan receptor B	0	0	1	1	Retina	Retinal progenitor proliferation	Chow et al., 1998, Mech Dev. 77(2):149-64.
	TGIF	TG-interacting factor	0	2	1	1	Retina	Retinal progenitor cell differentiation	Satoh et al., _2008, Exp Eye Res. 87(6):571-9.
	VSX2	Visual system homeobox 2	2	0	1	0	Retina	Eye organogenesis and retinal progenitor identity	Zou and Levine, _2012, PLoS Genet. 8(9):e1002924
	**TOTAL**		**12**	**9**	**14**	**10**			
**Brain development**	ATOH1	Atonal homolog 1 (Drosophila)	0	4	4	5	Cerebellum	Neurogenesis in the cerebellum	Mulvaney and Dabdoub A. 2012. J Assoc Res Otolaryngol. 13(3):281-93.
	BARHL2	BarH-like homeobox 2	0	5	3	8	Diencephalon	Neuroepithelial cell apoptosis	Juraver-Geslin et al., 2011, Proc Natl Acad Sci U S A. 108(6):2288-93.
	BHLHE22	Basic helix-loop-helix family, member e22	0	0	2	0	Neocortex	Stereotypic projection fates	Xu et al., 2002, Genomics 80: 311-318; Srinivasan et al., 2012, Proc Natl Acad Sci U S A. 109(47):19071-8
	BHLHE23	Basic helix-loop-helix family, member e23	0	0	2	0	Diencephalon	Diencephalic regionalization	Bramblett et al., 2002, Genomics. 79(3):402-12.
	CUX2	Cut-like homeobox 2	0	1	0	2	Cortex	Dendritogenesis	Cubelos et al., 2010, Neuron. 66(4):523-35.
	DMBX1	Diencephalon/mesencephalon homeobox 1	2	3	2	6	Brain	Midbrain and caudal diencephalon differentiation	Broccoli et al., 2002, Mech Dev. 114(1-2):219-23; Wong et al., 2010, BMC Dev Biol.10:100.
	DMRTA1	DMRT-like family A1	0	0	1	1	Cortex	Cortical neurogenesis	Kikkawa et al., 2013, Genes Cells. 18(8):636-49.
	EBF1	Early B-cell factor 1	2	2	2	2	Brain	Olfactory sensory neurons development	Davis and Reed, 1996, J Neurosci. 16(16):5082-94.
	Egr	Eiger	0	2	3	5	Brain	Neuronal programmed cell death	Kato et al., 2009, Development. 136(1):51-9.
	EGR1	Early growth response 1	6	10	7	20	Hindbrain	Motor and sensory systems neurones development	De and Turman, 2005, Arch Histol Cytol. 68(4):227-34.
	EGR4	Early growth response 4	0	0	1	3	Hippocampus	Neuronal differentiation	Crosby et al., 1992, Proc Natl Acad Sci U S A. 89(10):4739-43
	EMX2	Empty spiracles homeobox 2	2	0	1	0	Cortex	Cerebral cortex development	Spigoni et al., 2010, PLoS One. Jan 11;5(1)
	EN2	Engrailed homeobox 2	0	1	1	0	Cerebellum	Cerebellum foliation	Cheng et al., 2010, Development. 137(3):519-29.
	ESRRG	Estrogen-related receptor gamma	0	1	1	1	Spinal chord	Spinal chord motor neurones specification	Friese et al., 2009, Proc Natl Acad Sci U S A. 106(32):13588-93.
	FEV	FEV (ETS oncogene family)	2	4	0	0	Midbrain	Serotonin neuron precursors	Krueger and Deneris, 2008, J Neurosci. 28(48):12748-58.
	FOXB1	Forkhead box B1	0	1	2	1	Diencephalon	Axonal complexes histogenesis	Alvarez-Bolado et al., 2000, Development. 127(5):1029-38.
	GBX1	Gastrulation brain homeobox 1	0	1	1	0	Dorsal horn	Dorsal spinal cord development	John et al., 2005, Dev Dyn. 234(3):767-71.
	GBX2	Gastrulation brain homeobox 2	2	1	4	2	Brain	Forebrain and midbrain specification	Martinez-Barbera et al., 2001, Development.128(23):4789-800.
	GCM1	Glial cells missing homolog 1 (Drosophila)	0	0	1	2	Brain	Spinal cord development	Soustelle et al., 2007, Development. 134(3):625-34.
	GCM2	Glial cells missing homolog 2 (Drosophila)	0	0	1	1	Central nervous system	Gliogenesis	Soustelle and Giangrande, 2007, Dev Dyn. 236(8):2101-8.
	Hmx	H6-like-homeobox	0	1	1	0	Brain	Nervous system development	Wang et al., 2000, Mech Dev. 99(1-2):123-37
	IRF-8	Interferon regulatory factor 8	0	2	6	1	Brain and spinal chord	Apoptosis, inflammation and oxidative injury	Xiang et al., 2014, J Neurochem. 129(6):988-1001.
	KLF7	Kruppel-like factor 7 (ubiquitous)	0	1	0	0	Developing nervous system	Neuronal morphogenesis and axonal growth	Laub et al., 2005, Mol Cell Biol. 25(13):5699-711.
	LHX2	LIM homeobox 2	2	0	1	0	Telencephalon	Cortex development	Hébert and Fishell, 2008, Nat Rev Neurosci. 9(9):678-85.
	LHX3	LIM homeobox 3	0	3	4	1	Pituitary	Gonadotrope cells differentiation	Mullen et al., 2007, Mol Cell Endocrinol. 265-266:190-5; Savage et al., 2007, J Cell Physiol. 212(1):105-17.
	LHX3b	LIM class homeodomain transcription factor, Lhx3 subclass	0	0	1	0	Pituitary	Gonadotrope cells differentiation	Sloop et al., 2001, Gene. 265(1-2):61-9.
	NEUROD1	Neuronal differentiation 1	0	1	1	2	Brain	Neuronal differentiation	D'Amico et al., 2013, PLoS One. 8(6):e66487.
	NEUROD2	Neuronal differentiation 2	0	4	4	5	Brain	Neuronal differentiation	Wilke et al, 2012, Neural Dev. 7:9; Franklin et al., 2001, Child Neurol. 16(11):849-53
	NEUROG2	Neurogenin 2	0	0	2	0	Brain	Neuronal differentiation	Kovach et al., 2013, Cereb Cortex. 23(8):1884-900.
	OLIG1	Oligodendrocyte transcription factor 1	0	0	2	0	Brain	Oligodendrocyte repairing	Arnett et al., 2004, Science. 306(5704):2111-5.
	OLIG2	Oligodendrocyte lineage transcription factor 2	0	1	2	0	Brain	Oligodendrocyte specification	Matsumura et al., 2013, Neuropathology. 33(3):246-55.
	OLIG3	Oligodendrocyte transcription factor 3	0	4	2	2	Cerebellum	Neuronal development	Liu et al., 2008, J Neurosci. 28(40):10124-33.
	OTP	Orthopedia homeobox	2	1	1	0	Hindbrain	Dopaminergic neuronal specification	Fernandes et al., 2013, PLoS One. 8(9):e75002.
	OTX1	Orthodenticle homeobox	0	0	2	4	Ventral midbrain	Mesencephalic dopaminergic neuron development	Acampora et al., _2005, Brain Res Bull. 66(4-6):410-20; Simeone et al., 2009, Adv Exp Med Biol. 651:36-46.
	PHOX2A	Paired-like homeobox 2a	0	0	1	0	Hindbrain	Autonomic nervous system specification	Pattyn et al., 1997, Development. 124(20):4065-75.
	PHOX2B	Paired-like homeobox 2b	0	0	1	0	Hindbrain	Motor neuronal differentiatio	Dubreuil et al., 2000, Development. 127(23):5191-201.
	POU4F1	POU class 4 homeobox 1	0	0	1	0	Brain	neuronal growth	Latchman, 1998, Int J Biochem Cell Biol. 30(11):1153-7.
	PROP1	PROP paired-like homeobox 1	1	0	2	0	Pituitary	Somatotrope and gonadotrope cells differentiation	Pfäffle et al., 1999, Acta Paediatr Suppl. 88(433):33-41
	TFAM	Transcription factor A, mitochondrial	0	2	1	2	Cortex	Mitochondrial biogenesis	Yu et al., 2010, Neuroscience.169(1):23-38.
	unc-4	Protein UNC-4	0	1	1	0	Spinal cord	Synaptic movements	Schneider et al., 2012, Development. 139(12):2234-45.
	ZIC2	Zinc finger protein of the cerebellum 2	2	0	3	2	Cerebellum	Cell proliferation	Aruga et al., 2002, J Neurosci. 22(1):218-25.
	ZSCAN21	Zinc finger and SCAN domain containing 21	1	0	0	0	Cerebellum	Cell proliferation	Yang et al., 1999, Nat Genet. 22(4):327-35.
	**TOTAL**		**24**	**57**	**78**	**78**			
**Testis development**	Achi	Achintya	3	4	2	0	Testis	Spermatocyte meiosis	Perezgasga et al., 2004, Development. 131(8):1691-702.
	DMRT1	Doublesex and mab-3 related transcription factor 1	1	0	0	0	Testis	Sertoli cells and germ cells development	Agbor et al., 2013, Biol Reprod. 88(2):51.
	EMX2	Empty spiracles homeobox 2	2	0	1	0	Testis	Testicular determination	Ostrer et al., 2007, Sex Dev. 1(5):286-92.
	ETV5	ets variant 5	1	2	0	1	Testis	Spermatogonial stem cell self-renewal	Chen et al., 2005, Nature. 436(7053):1030-4.
	HSFY1	Heat shock transcription factor, Y-linked 1	0	2	0	1	Testis	Sperm function	Tessari et al., 2004, Mol Hum Reprod. 10(4):253-8.
	MYBL1	V-myb avian myeloblastosis viral oncogene homolog-like 1	0	1	1	3	Testis	Male germ cells meiosis	Toscani et al., 1997, Nature. 386(6626):713-7.
	NR2C2	Nuclear receptor subfamily 2, group C, member 2	4	10	9	13	Testis	Germ cell differentiation	Mu et al., 2006, Biochem Biophys Res Commun. 341(2):464-9.
	NR6A1	Nuclear receptor subfamily 6, group A, member 1	0	1	0	1	Testis	Spermatid nuclear elongation and condensation	Zhang et al., 1998, Mol Reprod Dev. 50(1):93-102.
	RFX4	Regulatory factor X, 4 (influences HLA class II expression)	0	0	1	0	Testis	Male haploid cells differentiation	Morotomi-Yano et al., 2002, J Biol Chem. 277(1):836-42; Kistler et al., 2009,Gene Expr Patterns.9(7):515-9.
	TWIST1	Twist family bHLH transcription factor 1	0	0	0	1	Testis	Germ cell tumor	Väre and Soini, 2010, APMIS. 118(9):640-7.
	WT1	Wilms tumor 1	0	5	9	12	Testis	Sertoli cells development	Bradford et al., 2009, Hum Mol Genet. 18(18):3429-38; Boyer et al., 2008 , Biol Reprod. 79(3):475-85.
	ZIM3	Zinc finger, imprinted 3	0	0	0	1	Testis	Testicular function	kim et al., 2001, Genomics. 77(1-2):91-8.
	**TOTAL**		**11**	**25**	**23**	**33**			
**Ovarian development**	AHR	Aryl hydrocarbon receptor	0	1	1	2	Ovary	Follicle growth and steroidogenesis	Hernández-Ochoa et al., 2009, Biochem Pharmacol. 77(4):547-59
	FIGLA	Folliculogenesis specific basic helix-loop-helix	0	0	0	2	Ovary	Folliculogenesis	Joshi et al., 2007, BMC Dev Biol. 7:67.
	FOXL2	Forkhead box L2	1	0	1	1	Ovary	Steroid metabolism	Benayoun et al., 2009, Adv Exp Med Biol. 665:207-26.
	hlh-29	Protein HLH-29	0	0	0	1	Ovary	Ovulation	White et al., Biology open. doi: 10.1242/_bio.2012046
	Obox2	Oocyte specific homeobox 2	0	0	0	1	Ovary	Oocyte development	Rajkovic et al., 2002, Genomics.79(5):711-7.
	OVO	CG6824 gene product from transcript CG6824-RE	7	4	3	5	Ovary	Germline formation and differentiation	Andrews et al., 2000, Development. 127(4):881-92.
	**TOTAL**		**8**	**5**	**5**	**12**			
**Testis & ovarian developme**	AR	Androgen receptor	6	20	8	11	Ovary/Testis	Gonad development and function	Zhou, 2010, J Androl. 31(3):235-43.
	ESR1	Estrogen receptor 1	2	6	2	4	Ovary/Testis	Ovulatory function, germ cell development	Hewitt and Korach, 2003, Reproduction. 125(2):143-9.
	ESR2	Estrogen receptor 2	3	4	3	2	Ovary/Testis	Ovulatory function, germ cell development	Hewitt and Korach, 2003, Reproduction. 125(2):143-9.
	NR0B1	Nuclear receptor subfamily 0, group B, member 1	0	1	1	1	Ovary/Testis	Gonad differentiation	Park et al., 2008, Biol Reprod. 79(6):1038-45.
	PR	Progesterone receptor	0	1	0	0	Ovary/Testis	Gonad development and function	Conneely et al., 2002, Recent Prog Horm Res. 57:339-55.
	**TOTAL**		**11**	**32**	**14**	**18**			
