# Supplementary material for: The pH sensitivity of Aqp0 channels in tetraploid and diploid teleosts
Source: FASEB J. 2015 Feb 9;29(5):2172–84. doi: 10.1096/fj.14-267625 (PMC4423293; doi:10.1096/fj.14-267625)
Supplement: Supplemental Data [file supp_fj.14-267625_Supplemental_Data1.doc]

**Supporting Information**

**Figure S1 Alignment of the UTR regions of Atlantic salmon *aqp0* mRNAs and primer design for qRT-PCR.** Nucleotide alignment of the 5’ and 3’ end UTR regions of *aqp0a1*, *-0a2*, *-0b1* and *-0b2*. The sequences of the primers used for qRT-PCR are shaded in dark blue for *aqp0a1*, palid blue for *aqp0a2*, red for *aqp0b1*, and green for *aqp0b2*. The nucleotide substitutions between paralogs are indicated in red.

(TIF)

**Table S1 Potential binding sites of relevant transcription factors (TFs) identified in the 5’ flanking region of Atlantic salmon *aqp0* genes.** The table lists the number of TF binding sites in the 5’ flanking region with an score >0.9 identified with the TRANSFAC 7.0 software. Only TFs known to be involved in lens, eye, brain or gonad development are indicated.

(PDF)
