# Supplementary material for: The pH sensitivity of Aqp0 channels in tetraploid and diploid teleosts
Source: FASEB J. 2015 Feb 9;29(5):2172–84. doi: 10.1096/fj.14-267625 (PMC4423293; doi:10.1096/fj.14-267625)
Supplement: Supplemental Data [file supp_fj.14-267625_Supplemental_Figure1.pdf]

Supplemental Figure S1

A

5’ end UTR

```
aqp0a1    -146 CTTTGAAGTTTGCTCTCTTGTTTTTT---TATCCTACCTTGAAGGCATCA-----------------------CTGAGAT--AGTCATTA
aqp0a2    -181 CTTTTAAGTTTGCTGTCTTGTTTTTTGTTTATACTACCTTGAAGGCATCA-----------------------CTGAGAT--AGTCATTA
aqp0b1    -173 CGTTTGGGTCAGC---CCAGCAAACTG-----ACCTCCAAAGGGATAAAA---------------------AAAAAAAATTAAGTG----
aqp0b2    -186 CGTTTAGGTCAGC---CCAGCTAGCTG-----ACTGCCAAAGGGAAATCACTTTTTTTTTTTTTTTTTTGCATCTGAAAC--AGTG----
               * **   ** **   *  *     *       *  **     *  *  *                         * *   ***

aqp0a1     -89 TTGATACGCTGAGCAGAGCTAGCTCACTCTAAAGGAGAATACACATTT---------GGGGTAGC---------CAT-------------
aqp0a2    -116 TTGATACGGTAAACAGAGCTTGGTGACTCAAAAGGAGAATACACATTTT---CTCCTGTTTTCGCTCCTCTCTTCATCCCTCCATTGC-
aqp0b1    -116 -CAAAACGAGTAACAAGACT------TTTAAAACG----TACACGCTTGCCTTTTCTAGATTCAC----CTTGACAACTTTTTGTTGTT
aqp0b2    -111 -CAAAAAAATCACCTAGACT------TTTAGAAGG----TACAAGCTT----TTTCTAGATTCAC----CCCAACAACTTTTTTCCC--
                  * *     * *    **       *   ** *     ****   **             *  *

aqp0a1     -30 --TTGGGCTAGCCTCTTGACTTTT-----------------TGGCCACC  -1
aqp0a2     -31 -TTTGGGGTAGCATCTTGACTTTT-----------------TGGCCACC  -1
aqp0b1     -42 GTTGAGAAAAACATTTTCTGTTACAAGGAGTG-------AACAGCCAGC  -1
aqp0b2     -43 ------AAGAACATTTTCCGTTCTGAGGAGCGTCCAGTAAACGGCCAGC  -1
                        * * * **   **                      **** *
```

B

3’ end UTR

```
aqp0a1     793 GCCTGG---TGAAGAAGGTTGGCTTTCTGAACCCCACATTCCCCTATTCCGTACCCAAACCTCCTCCGAACCCTCAACCCTACCCAACA
aqp0a2     793 GCCTGG---TGGAAGA------------------------------------------------------------------------
aqp0b1     793 ACCTGCACCTGTCTGG------------------------------------------------------------------------
                ****    **

aqp0a1     879 CACATACACACACATTACTCTTCCTCTCTAACCCACACACCCACATAGTACACACAGACCACCACGACAACTCCACCTTTCTCACCCTC
aqp0a2         -----------------------------------------------------------------------------------------
aqp0b1         -----------------------------------------------------------------------------------------

aqp0a1     968 CTCATCCTTACTCTGCTGATCCAGAAACAGTACATGCTGAGTTACTGGGAAGCCAAAGCCCTAACCCTCCTCCGACCTGTGAACTATGA
aqp0a2     806 ----------------------AGAAACAGGACATGCTGAATTACAGGGCAGCCAAAGCCCTAACCACCCTCCAACACTCCACCACTGA
aqp0b1     809 ----------------------GGACAAGGGACGAG----------GGACAG-----GCCCTG--TGTCCGCCAAC-CCCCA-CGCTGG
                                      ** *  * **  *           **  **     *****      ** ** **     * *  **

aqp0a1    1057 CCCC--------CTCCCCCCCTTGGACTGCAGAACAAAGATGGAGGGTAAGAAGAGGACTTAGTTGC-CACTGAAGAAAGCACCCTGCT
aqp0a2     873 CCCTTACCCATACCTCCCCCCTTGGACTGCAGGATAAAGATGGAGGGTAAGAAGAGGACTTAGTTGC-CACTGAAGAAAGCACCCTGCT
aqp0b1     857 CCC------------CATCCCATGAGC------ACATATACAGTCCCT----------CTCATTGACTCACTAACTAACACACACACCC
                ***            *  *** **  *     * * *  *  *    *          ** * *  * **** *  **  *** *  *

aqp0a1    1137 GCGGC-CTGTTGTGTAGGGAGTGGGTGACTCCTGGCTGACCAGGTGGCTGTAACTGCTCAGGACAGGGGCATCTCTCTCTCTTCTTTTC
aqp0a2     961 GTGGC-CTGTGGTGTAGGGAATGGGTCACTCCTGGCTGACCAGGTGGCTATAACTGCTCAGGACAGGGGCATCTCTCTCTCT------C
aqp0b1     918 ATGTCGCTACGA--CAGGCGGCCAGTGA-----GGGCAAGCGAGCAAATCTGA--GTT---------GATGTTTGACTGCCT-------
                 *   **       ****     ** *     **   *  *  *    * * *  * *         *   * *  **  **

aqp0a1    1225 TCTCTATATTTTG-TAGTCTGCTAGGAGTGAAGCTACAGTAGAGCAGTGAACCTCATGCTTTTTCCATTTTTTATGAAACTTCTGGTAC
aqp0a2    1043 TCTCT-TGTTT-G-TAGTCTGCTAGGAGTGAAGCTACAGTAGAGCAGTGGACTTC-----------------------------------
aqp0b1     982 TCGCCAACTTGAGATCGTCCGT--AGCTTAGAGCTAGATCTGTATGGTGGATTCC-----------------------------------
               ** *    **  * * *** *    *  *  ***** *   *    *** *   *

aqp0a1    1303 CTAATGTGCATGTTATTATTATGCCCTTGAAGAATTTATGAGGATTTTTTTCAACTCTAGTTATATTTTCAAGTCATGCATTGTGTTGT
aqp0a2    1095 ------TGCATG-------------CTT------TTTA-----ATTTTTTTCA--TCCAGTTTGAATCTC------TCCCTTGTGTT--
aqp0b1    1035 --------TCTG----------------------TCA-----ACTTTTTTAA----CTGTTTG--TCTT------CCAACTG------
                         **                       * *      * ****** *      ***    * *            **

aqp0a1    1402 TTGTGCGTGTTTTAGAAGACCATTGTTATTTTTATTT  1438
aqp0a2    1144 ----------------------TTATTGTTTTTGTTT  1158
aqp0b1    1070 ------------------------ATTGGCTAAGTAA  1082
                                        **   *   *
```
